# Association between Periodontal Disease and Systemic Inflammatory Conditions Using Electronic Health Records: A Pilot Study

**DOI:** 10.3390/antibiotics10040386

**Published:** 2021-04-04

**Authors:** Georgios S. Chatzopoulos, Alejandro Cisneros, Miguel Sanchez, Larry F. Wolff

**Affiliations:** Division of Periodontology, Department of Developmental and Surgical Sciences, School of Dentistry, University of Minnesota, Minneapolis, MN 55455, USA; cisne025@umn.edu (A.C.); sanch272@umn.edu (M.S.); wolff001@umn.edu (L.F.W.)

**Keywords:** epidemiology, inflammation, oral-systemic disease, periodontal disease, tooth loss

## Abstract

Aims: To investigate the association between periodontal disease and systemic inflammatory conditions and examine the link between medical conditions and the extent of missing teeth in a large population. Methods: In this retrospective study, a total of 4890 randomly selected patients who had attended the University of Minnesota dental clinics were analyzed. Severity of periodontal disease was determined based on the percentage of bone loss, evaluated through the examination of a full-mouth intraoral series of radiographs. The number of missing teeth was calculated from the examined radiographs, while ten systemic inflammatory conditions were extracted from patients’ self-reported medical histories. Results: Moderate bone loss was observed in 730 (14.9%) and severe in 323 (6.6%) patients of the total population, while the mean number of missing teeth was 3.54 ± 3.93. The prevalence of systemic conditions and tobacco use were gender-dependent (*p* < 0.05). Regression analysis showed that hypertension, arthritis, asthma, diabetes and HIV were associated significantly with the severity of bone loss, while diabetes and lupus with the extent of missing teeth. Conclusions: The findings reported in our study add to this body of knowledge, strengthening the association between periodontal disease with systemic inflammatory conditions.

## 1. Introduction

As early as 400 BC, Hippocrates suggested that tooth extraction might cure arthritis, and Miller in 1891 stated that infections in the mouth could lead to pathology at distant sites of the body [1]. At the end of the 20th century, studies showed that simple oral hygiene habits can translocate bacteria, toxins and inflammatory molecules to other sites of the body (focal theory of infection), resulting in a higher risk of bacteremia for individuals with an oral infection [2]. Immunocompromised individuals such as diabetics may exhibit susceptibility to disease because of their lower ability to eliminate the transient bacteremia in the circulation.

Williams and Offenbacher (2000) proposed the term “periodontal medicine,” aiming to point out the significant effect of systemic diseases on the periodontium [3]. Periodontal disease has been associated with a number of systemic conditions, including diabetes mellitus, cardiovascular, cerebrovascular, respiratory diseases, preterm birth and low birth weight [3]. This relationship between periodontal disease and systemic conditions is widely described as being “bidirectional,” but further research is still needed to establish causality. For the most part, the biological connection between periodontal disease and systemic conditions is still unknown. However, the systemic spread of bacteria, bacterial toxins and bacterial products from the oral cavity, as well as their ability to travel to distant sites, may adversely impact the development of a systemic disease or conditions. This mechanism is referred to as metastatic infection, which comes from the Greek word “*μετάστασις”* (metastasis), meaning displacement [4,5]. Another theory proposes that periodontal disease triggers a chronic inflammatory cascade through inflammation and inflammatory mediators that may link periodontitis to systemic afflictions [5]. In addition, the expression of virulence factors produced by periodontopathogens and the presence of pathogens in nonoral tissues may support the biological relationship between chronic oral diseases such as periodontal disease and systemic diseases [5].

Periodontal disease is part of a larger group of inflammatory diseases, and their association may be attributed to common inflammatory mechanisms and/or a disruptive host immune response that affects the oral cavity as well as other areas in the human organs [6]. Microbial dysbiosis does not always precipitate periodontitis, but it could initiate a state of disease in patients with susceptible hosts such as patients with systemic medical conditions. Systemic diseases can unfavorably affect the homeostatic balance and lead to tissue destruction. Identifying the associations between periodontal disease and systemic medical conditions can provide a holistic approach to patient care management. Although a number of studies have been conducted in the past, the small sample sizes, the high heterogeneity and the increased risk of selection bias may have skewed the results. Currently, the use of electronic health records can be used as an effective and reliable method for mining associations between periodontal and medical conditions. Therefore, the aim of this study was to investigate the association between periodontal disease and systemic inflammatory conditions and examine the link between medical conditions and the extent of missing teeth in a large population

## 2. Materials and Methods

### 2.1. Sample

In this retrospective chart review study, a total of 10,201 dental records were retrieved from the electronic database at the University of Minnesota School of Dentistry, while 5000 dental charts were randomly selected by a computer-generated program and evaluated from patients who had presented to the School of Dentistry clinics for treatment from 2012 to 2016. The included dental records of patients with periodontal and restorative dental needs were retrieved from different dental clinics at the University of Minnesota School of Dentistry (Predoctoral and Graduate clinics). Dental charts were randomly selected to allow the generalizability of the findings and to eliminate bias in the patient selection process. The protocol of the school prior to any dental procedure is to verbally follow-up and update the medical history (patients’ medical history). In regard to the radiographic evaluation, each patient’s radiographs were examined to determine the percentage of bone loss as well as the number of missing teeth. Records of patients that received any type of dental treatment (primarily periodontal and restorative treatments) were included in the analysis, but patients had to be at least 18 years of age, have six or more remaining teeth, a full set of periapical radiographs and completion of the dental school medical questionnaire. The study was approved by the Institutional Review Board for medical record chart review at the University of Minnesota School of Dentistry (No. 1603M85323).

### 2.2. Data Extraction

Relevant data were obtained from the dental charts, radiographs and medical histories of patients who met the inclusion criteria of the study. Three independent evaluators reviewed the full-mouth series of radiographs and medical histories of patients who presented to the School of Dentistry for treatment. Datasheets were created for all available electronic dental charts that had a digital full-mouth series of radiographs taken in the school’s radiology department at the University of Minnesota School of Dentistry (*n* = 10,201), including chart number, date of birth, age at the time of the radiographs, gender, tobacco use and all examined medical conditions. Inflammatory systemic diseases, including arthritis, asthma, cancer, diabetes, HIV, hepatitis, hypertension, irritable bowel syndrome, lupus, and Parkinson’s disease, were recorded and analyzed due to their systemic inflammatory burden. The included diseases/conditions are examples of diseases/conditions mediated by chronic inflammation, as reported by the Centers for Disease Control and Prevention (www.cdc.gov using the keyword inflammation, accessed on 17 October 2018). Seventy-seven dental records were excluded due to an incomplete medical history, 300 were excluded to age limitation and the remaining 9824 records were randomized. Five thousand records were then selected for the final evaluation of the digital full-mouth series of radiographs in regard to the percentage of bone loss and the number of missing teeth as well as for an evaluation of each patient’s medical history. Severity of periodontal disease was determined based on the percentage of bone loss. Bone loss was a measure of the distance between the cementoenamel junction (CEJ) and the alveolar crest for the entire dentition (all present teeth). All bone loss measurements were taken on both interproximal sides of each present tooth, apart from third molars. A patient’s bone loss was categorized after taking into consideration the normal bone height of 1–2 mm between the alveolar bone and the CEJ [7]:

(1) None/mild bone loss when the examined individuals presented generalized (>30% of the teeth) bone loss of ≤25%.
(2) Moderate bone loss when the examined individuals exhibited generalized (>30% of the teeth) bone loss of 26–50%.
(3) Severe bone loss when the examined individuals showed generalized (>30% of the teeth) bone loss of >50% or ≥4 posterior teeth (premolars and/or molars) with >50% bone loss.

The number of missing teeth was calculated from the examined radiographs by subtracting the total number of teeth present on the radiographs from 28. 

### 2.3. Inter-/Intraexaminer Calibration

The inter- and intraexaminer reliability was performed to determine the degree of agreement among and within the three independent examiners in regard to the percentage of bone loss and the number of missing teeth. Two hundred dental charts evaluated by each examiner were randomly selected and were re-evaluated to assess the agreement between the two readings. In addition, the first evaluator (G.S.C.) examined 10 dental charts out of every one hundred evaluated records from the other two examiners (A.C., M.S.) to monitor the consistency of the percentage of bone loss and the number of missing teeth among the examiners. The Kappa index of agreement for the number of missing teeth ranged from 0.88 to 0.97 and for the severity of bone loss from 0.93 to 0.95, which can be considered excellent agreement [8].

### 2.4. Statistical Analysis

A sample size calculation was performed prior to the initiation of the study. Anticipating an effect size (f^2^) of 0.02 in a multiple regression model of 13 predictors, the required number of patients with a power of 90% and a significance level of 5% was calculated to be 1135 patients. This retrospective chart review study included a total of 4890 individuals and therefore is powered to reach sound conclusions in regard to the examined associations between periodontal disease and systemic medical conditions.

The data from the included dental charts such as demographic characteristics, medical history, tobacco use, bone loss and number of missing teeth were collected and recorded in a computer database using Excel and analyzed using statistical software. Descriptive statistics, including frequencies, means, standard deviations and ranges, were reported. A chi-square test was utilized to assess the prevalence of the medical conditions between females and males. The association between systemic diseases and the percentage of bone loss groups (none to mild, moderate, severe) were assessed with Fisher’s exact test (univariate analysis) and adjusted for age, gender, smoking and diabetes with logistic regression analysis for the parameters that reached statistical significance in the univariate analysis. The association between the systemic inflammatory medical conditions and the number of missing teeth was examined with logistic regression analysis, and for the conditions that were statistically significantly associated with missing teeth, adjusted logistic regression analysis for age, gender, smoking and diabetes was completed. The odds ratios and 95% confidence intervals were calculated for the systemic medical conditions that were included in the multivariable regression. All tests of significance were evaluated at the 0.05 error level with a statistical software program (SPSS v.19.0, IBM, Armonk, NY, USA).

## 3. Results

### 3.1. Patient Records Included and Population Characteristics

**Figure 1:**
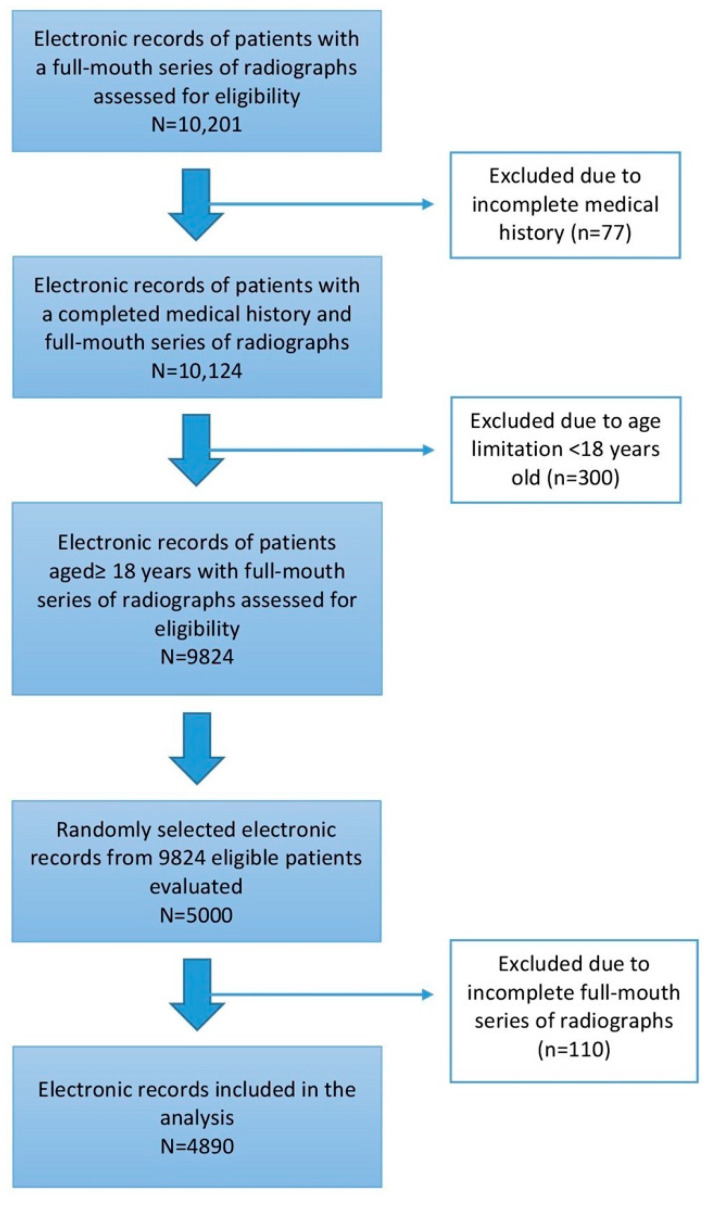
Detailed information about patients’ eligibility and the process of assessment for inclusion and exclusion.

Detailed information about patients’ eligibility and the process of assessment for inclusion and exclusion for this study is shown in a flow diagram (Figure 1). Out of the 10,201 initially screened charts, 77 and 300 records were excluded from the analysis due to an incomplete medical history and age limitation, respectively. A total of 9824 patients remained eligible, and from these, 5000 randomly selected patient records were evaluated. From these 5000 patients, 110 records had an incomplete full-mouth series of radiographs. Thus, a total of 4890 patients were included in the final analysis to investigate the association between periodontal disease/bone loss and systemic diseases as well as tobacco use.

Population characteristics compared between the radiographic bone loss groups are presented in Table 1. None to mild bone loss was observed in 3837 individuals (78.5%), moderate in 730 (14.9%) and severe in 323 (6.6%). The mean age of the included subjects was 54.06 (±17.85, range from 18 to 94), and 52.7% of the population were males. The mean number of missing teeth in the total population was 3.54 ± 3.93 (range from 0 to 22). Patients with moderate and severe bone loss were older and exhibited a higher number of missing teeth than those with none to mild bone loss (*p* < 0.001). Post-hoc Tukey HSD tests showed that patients diagnosed with severe bone loss exhibited significantly more missing teeth than those with none to mild (*p* < 0.001) and moderate (*p* = 0.007) bone loss. Post-hoc analysis with Tukey test also revealed that individuals with moderate bone loss were significantly older than those categorized with none to mild (*p* < 0.001) and severe (*p* < 0.001) bone loss. Male patients and tobacco users were also diagnosed with severe bone loss significantly more frequently than females and tobacco nonusers (*p* < 0.001).

### 3.2. Prevalence of Self-Reported Systemic Conditions

The prevalence of the examined self-reported systemic conditions for all patients compared between females and males are presented separately for females and males in Table 2. Hypertension (29.1%), arthritis (21.6%) and diabetes (11.1%) were found to be the most prevalent systemic conditions, while lupus (0.5%), HIV (0.5%) and Parkinson’s disease (0.3%) were rarely observed. A number of systemic diseases were found to be statistically significantly more prevalent in females compared to males including arthritis, asthma, irritable bowel syndrome and lupus (*p* < 0.001). On the other hand, hypertension, diabetes and HIV were significantly more prevalent in males than in females (*p* < 0.05).

### 3.3. Self-Reported Systemic Conditions Associated with Severity of Bone Loss

Self-reported systemic inflammatory diseases associated with moderate and severe periodontal disease are shown in Table 3. In the univariate unadjusted analysis, five medical conditions showed a significant association with the percentage of bone loss. Hypertension (*p* < 0.001), arthritis (*p* < 0.001), diabetes (*p* = 0.0006) and HIV positive/AIDS (*p* = 0.009) were significantly more prevalent in individuals with moderate and severe bone loss than those with none to mild bone loss. Regression analysis after adjusting for age, gender and smoking associating the percentage of bone loss with systemic medical conditions was also performed for the conditions that showed a significant association (*p* < 0.05) in the univariate unadjusted analysis. The results are shown in Table 4. All five medical conditions remained significant (*p* < 0.05) in the adjusted analysis. These included arthritis (*p* = 0.004), asthma (*p* < 0.001), diabetes (*p* < 0.001), HIV positive/AIDS (*p* = 0.005) and hypertension (*p* < 0.001).

### 3.4. Self-Reported Systemic Conditions Associated with Missing Teeth

The unadjusted analysis in determining the association between the number of missing teeth with self-reported systemic medical conditions showed that four conditions were significantly associated. More specifically, arthritis (*p* < 0.001), diabetes (*p* < 0.001), hypertension (*p* < 0.001) and lupus (*p* = 0.001) displayed a significant association with the number of missing teeth. After adjusting for age, gender and smoking, only diabetes (*p* < 0.001) and lupus (*p* = 0.004) remained statistically significant.

## 4. Discussion

This study was performed to evaluate the association between periodontal disease and the extent of missing teeth with the presence of systemic inflammatory medical conditions in a large cohort seeking dental care at the University of Minnesota School of Dentistry. Moreover, we comparatively evaluated the severity of bone loss and the number of missing teeth from a full-mouth series of radiographs with a self-reported medical history recorded on an electronic database. Randomly selected patient data related to a total of 4890 patients were retrospectively analyzed, and it was hypothesized that systemic medical conditions with an inflammatory background lead to periodontal disease and tooth loss. More specifically, individuals with a compromised medical history will demonstrate a greater amount of bone loss and a greater number of missing teeth than patients without a significant medical history. The results of the present study indicated that (i) the prevalence of systemic inflammatory conditions was gender-dependent; (ii) hypertension, asthma, arthritis, diabetes and HIV were associated significantly with alveolar bone loss; (iii) and diabetes and lupus were significantly associated with the extent of missing teeth. The evaluation of the data in this investigation was to determine where trends existed with the plan to subsequently evaluate a much larger data set.

In the current study of individuals with different medical conditions and numbers of missing teeth, the prevalence of severe bone loss was 6.6%, and the prevalence of disease was significantly associated with the gender and age of the examined population. Older individuals and males were diagnosed with severe bone loss significantly more frequently than younger individuals and females, respectively (*p* < 0.05). Individuals diagnosed with severe periodontal disease (severe bone loss group) exhibited a higher number of missing teeth than those categorized into the none/mild bone loss group (*p* < 0.001). In addition, smoking habits were significantly associated with the severity of bone loss (*p* < 0.001). In particular, patients in the severe bone loss group were significantly (*p* < 0.001) more likely to smoke (30%) than individuals with none to mild (13.1%) and moderate (18.1%) bone loss. These findings agree with previous research that has demonstrated the negative impact of smoking on periodontal disease. Tobacco use is a major environmental risk factor for periodontal disease that has shown a two- to eight-fold increased risk for clinical attachment and bone loss, which is higher in heavy smokers when compared to light smokers [9,10,11]. Arthritis was significantly associated with bone loss (*p* = 0.004). Both chronic inflammatory diseases are characterized by excessive tissue destruction such as bone, cartilage and other periarticular tissues in rheumatoid arthritis and alveolar bone, periodontal ligament and gingiva in chronic periodontal disease, which result from the activation of cytokine-driven osteoclasts [12]. This association may be attributed to multiple biologically plausible mechanisms that include the activation of underlying and overlapping systemic inflammation [13].

Asthma was significantly associated with bone loss in the present study (*p* < 0.001). An association between asthma and periodontal disease has been reported in previous studies that showed asthmatic patients included in a population-based retrospective cohort study being at an increased risk of developing periodontal diseases [14]. Asthmatic individuals are associated with gingivitis, periodontal inflammation, plaque accumulation, reduced salivary flow and an increased risk of caries than nonasthmatic subjects [15,16,17]. This finding may be attributed to mouth breathing habits and the use of inhalational medications from patients with severe asthma [18]. In the present investigation, asthma demonstrated a protective effect for inflammation (OR (95% CI): 0.695 (0.564–0.857)). This could be attributed to the anti-inflammatory medications that are regularly used by asthmatic patients. Due to the lack of data on individuals’ medications, no analysis was carried out to examine this potential association. Type 2 diabetics with poor glycemic control are more prone to alveolar bone loss as well as a higher risk for disease progression compared to well-controlled diabetics and nondiabetics [19,20]. Diabetes showed a statistically significant association with bone loss (*p* < 0.001) and the extent of missing teeth (*p* < 0.001), as shown after adjusting for age, gender and smoking. This might be attributed to the increased systemic inflammation as well as the robust inflammatory reaction to microbial challenge in diabetics that results in accelerated bone loss [21].

In our study, regression analysis revealed that HIV was significantly associated with bone loss (*p* = 0.005). The decreased monocyte chemotaxis and phagocytosis, the increased production of TNF-alpha, IL-4, IL-10 and IL-1, as well as the increased susceptibility to infections and the delayed healing response, explain the susceptibility of HIV-positive individuals to periodontal disease [22,23,24,25]. In addition, the loss of bone tissue in HIV is multifactorial [26]. HIV-infected individuals demonstrate alterations in bone metabolism and bone mineral density, as well as a higher risk of developing osteopenia and osteoporosis, hypogonadism and vitamin D deficiency [26]. The common hepatitis C coinfection may also increase the risk of low bone mineral density, and antiretroviral therapy leads to bone turnover increases [26,27]. Although previous research has shown an increase in the prevalence of periodontal manifestations in relation to HIV infection despite the use of highly active antiretroviral therapy, more recent highly powered research revealed that this association may be explained by confounding factors such as smoking [28]. The severity of periodontal disease is not related to HIV infection [29].

Periodontal disease may ultimately lead to tooth loss if left untreated, and historically, tooth loss has been used as a marker for periodontal disease [30]. We hypothesized that the association between periodontal disease through the evaluation of bone loss with systemic medical conditions will also be reflected by the number of missing teeth. Out of the 10 examined medical conditions, four of them were significantly associated with the number of missing teeth in the unadjusted analysis (*p* ≤ 0.05). Hypertension, arthritis, diabetes and lupus were all statistically significantly associated with tooth loss. After adjusting for confounding factors, diabetes *p* < 0.001) and lupus (*p* = 0.004) maintained the significance. It is noteworthy that periodontal disease is not the only cause of tooth loss, but caries, failed root canal treatment, tooth fracture or other potential reasons may contribute to loss of a tooth. The design of the present study did not enable us to determine the reason for the loss of a missing tooth. In an analysis of data from the National Health and Nutrition Examination Survey (NHANES), diabetics over 50 years of age were associated with a greater number of missing teeth and were more likely to be edentulous rather than the nondiabetics [31]. In another study that utilized data from the Behavioral Risk Factor Surveillance System (BRFSS), diabetic individuals between 18 and 44 years old exhibited a strong association with the number of missing teeth [32].

An association between periodontal disease and systemic autoimmune diseases such as systemic lupus erythematosus has been reported. Both periodontal disease and systemic lupus erythematosus share similar mechanisms of an immune response that lead to tissue damage with the destruction of periodontal tissues in periodontal disease and destruction of connective tissues in systemic lupus [33]. Systemic lupus significantly adversely affects periodontal health due to increased local inflammation and altered subgingival microbiota that is characterized by dysbiosis, higher subgingival bacterial load and greater proportions of pathogenic bacteria, as well as reduced microbial diversity [34]. Oral health and periodontal disease have been investigated for their potential impact on atherosclerotic cerebrovascular diseases such as stroke [35]. Multiple studies have reported an association of tooth loss with ischemic stroke [36]. In particular, individuals with fewer teeth or edentulism have shown an increased odds ratio for stroke independently of the well-established risk factors such as gender, age, education, smoking and body mass index [36]. Biologically plausible mechanisms that support this association are the bacterial challenge that leads to chronic inflammation, the increased inflammatory response and the high production of proinflammatory cytokines [37]. The compromised mastication due to tooth loss disrupts normal chewing ability and may affect brain function and lead to ischemic stroke [38,39].

Self-reported medical history was utilized in the present study to determine the prevalence of a number of systemic medical conditions and their association with periodontal disease. The retrospective design of the study did not allow us to include laboratory and/or physical examination, but self-reported data are commonly used in research and are considered valid and economical [40]. Limitations related to self-reported data are associated with social desirability bias in order to avoid criticism as well as patients’ difficulty understanding health care information [41]. In spite of the limitations, self-reported data can be used accurately for chronic systemic diseases and may be a feasible option for routine monitoring of chronic conditions to allow for prevalence measures in a large population [42]. A limitation of the study is the lack of clinical information about soft tissue examination and clinical periodontal measurements. These data would be used to distinguish between plaque-induced and non-plaque-induced lesions [43,44].

A number of medical conditions demonstrated no dose-dependent effect, which may be explained by the time a medical condition occurs and when it is diagnosed. Other possible explanations may be the presence of confounding factors or other biases that disrupt a dose-dependent response; for example, bone loss was assessed as a categorical variable because the included patients were classified into three groups (none to mild, moderate, severe bone loss) based on the percentage of bone loss. Assessing bone loss as a continuous variable may show a dose-dependent effect of the examined medical conditions.

In the present investigation, the amount of bone loss was calculated based on three reference points: the cementoenamel junction (CEJ), the alveolar bone crest and the root apex. The distance between the CEJ and the alveolar bone crest as well as between the CEJ and the root apex (root length) were measured. The percentage of bone loss was calculated as the ratio of the difference between those two distances and the root length multiplied by 100. Then, it equated the severity of bone loss as none to mild (0–25%), moderate (26–50%) and severe (>50%) after considering the normal bone height, which is 1–2 mm below the CEJ. The radiographic measurements were performed interproximally between all present teeth in the dentition (full-mouth protocol), apart from third molars, to avoid misclassification bias due to the use of partial-mouth protocols that may result in inaccurate periodontitis-systemic disease associations [45]. An inherent limitation of standard radiographs is that 30–50% of the bone mass must be lost before detecting it directly by eye [46]. Therefore, early signs of bone loss will not be detected until there is a 30–50% bone demineralization, which underestimates the radiographically diagnosed periodontitis. To overcome this limitation and minimize the risk of underestimating the diagnoses, individuals with 0–25% bone loss were categorized into the “the none to mild bone loss” group. In addition, due to the retrospective study design, standardization of the intraoral radiographs was not possible and may be considered a limitation of the study.

Today, the relationship between periodontitis and other pathological systemic conditions has been suggested in numerous epidemiological studies. The unbalance of the oral and gut microbiota may negatively impact systemic health due to different metabolic interactions that lead to dysbiosis [47]. Diabetes mellitus, cardiovascular disease, osteoporosis, respiratory disease, dementia, rheumatoid arthritis and cancer have all been associated with periodontal disease and inflammation. Periodontal disease and inflammation are thought to play an important role as the underlying common mechanism for these systemic disease conditions [48,49,50]. A hyperinflammatory neutrophil phenotype has been linked to the pathogenesis of chronic periodontal disease and systemic diseases related to periodontitis such as diabetes and cardiovascular disease [49,50,51,52]. This finding suggests that common susceptibility may contribute cumulatively to comorbidity.

## 5. Conclusions

A growing body of evidence in the scientific literature over the past two decades strongly suggests an association between periodontal disease and some systemic conditions as well as tobacco use. The findings reported in our study add to this body of knowledge, strengthening the association between periodontal disease with systemic inflammatory conditions. Further prospective or longitudinal investigations are needed to rule out confounding factors as a possible explanation for the association between periodontal and the various systemic diseases. Oral health is part of general health, and the findings of the present study show that periodontal disease is associated with systemic inflammatory conditions, possibly due to an underlying disruption of the inflammatory homeostasis. The cooperation between medical and dental professionals is critical to reduce the prevalence of periodontal disease and its comorbidities.

## Figures and Tables

**Table 1:** Characteristics of the patient cohort and comparison between the bone loss groups.

	Total Population (N = 4890)	Bone Loss			*p*-Value *
		None to Mild (*n* = 3837–78.5%)	Moderate (*n* = 730–14.9%)	Severe (*n* = 323–6.6%)	
Age (mean ± SD) in years	54.06 ± 17.85	51.72 ± 18.23	63.86 ± 13.21	59.61 ± 13.05	**<0.001 ^†^**
Males (%)	52.7	51.2	55.2	63.8	**<0.001 ^‡^**
Missing teeth (mean ± SD)	3.54 ± 3.93	2.87 ± 3.47	5.77 ± 4.43	6.52 ± 4.67	**<0.001 ^†^**
Tobacco use (%)	14.9	13.1	18.1	30.0	**<0.001 ^‡^**

* Level of significance: *p* < 0.001. ^†^ For continuous variables (age and missing teeth), ANOVA was utilized. Bold values represent a statistically significant difference. Patients with moderate and severe bone loss when compared to “none to mild” bone loss were older and exhibited a higher number of missing teeth (*p* < 0.001). ^‡^ For gender and tobacco use, a chi-square test was used. Bold values represent statistically significant differences. Male patients and tobacco users were diagnosed with severe bone loss significantly more frequently than females and nontobacco users (*p* < 0.001).

**Table 2:** Occurrence of examined systemic conditions for all patients compared between females and males.

Self-Reported Systemic Inflammatory Diseases	Total (*n* = 4890)	Males (*n* = 2575)	Females (*n* = 2315)	*p*-Value *
Hypertension	29.1	31.9	25.9	**<0.001**
Arthritis	21.6	17.2	26.3	**<0.001**
Asthma	11.1	6.1	11.1	**<0.001**
Diabetes	10.6	11.7	9.3	**<0.001**
Cancer	9.1	8.9	9.5	0.49
Irritable bowel syndrome	3.9	2.0	6.0	**<0.001**
Hepatitis	2.0	2.3	1.6	0.06
Lupus	0.5	0.0	1.0	**<0.001**
HIV positive/AIDS	0.5	0.9	0.1	**<0.001**
Parkinson’s disease	0.3	0.4	0.2	0.19

* Chi-square test was used to compare males and females in regard to the occurrence of systemic conditions. Bold values represent statistically significant differences with *p* ≤ 0.05.

**Table 3:** Occurrence of self-reported systemic inflammatory diseases in the patient cohort and bone loss groups.

Self-Reported Systemic Inflammatory Diseases	Bone Loss				*p*-Value
	Total Population (*n* = 4890)	None to Mild (*n* = 3837–78.5%)	Moderate (*n* = 730–14.9%)	Severe (*n* = 323–6.6%)	
Hypertension	1421 (29.1)	1000 (26.1)	291 (39.9)	130 (40.2)	<0.001
Arthritis	1054 (21.6)	777 (20.3)	209 (28.6)	68 (21.1)	<0.001
Asthma	415 (8.5)	355 (9.3)	42 (5.8)	18 (5.6)	0.001
Diabetes	516 (10.6)	348 (9.1)	115 (15.8)	53 (16.4)	0.006
Cancer	447 (9.1)	338 (8.8)	83 (11.4)	26 (8.0)	0.07
Irritable bowel syndrome	191 (3.9)	158 (4.1)	26 (3.6)	7 (2.2)	0.19
Hepatitis	96 (2.0)	70 (1.8)	21 (2.9)	5 (1.5)	0.15
Lupus	23 (0.5)	16 (0.4)	3 (0.4)	4 (1.2)	0.11
HIV positive/AIDS	26 (0.5)	14 (0.4)	8 (1.1)	4 (1.2)	0.009
Parkinson’s disease	14 (0.3)	8 (0.2)	5 (0.7)	1 (0.3)	0.09

**Table 4:** Adjusted analysis for the association between bone loss and self-reported systemic medical conditions/tobacco use.

Self-Reported Systemic Inflammatory Diseases	Adjusted Odds Ratio * (95% Confidence Interval)	*p*-Value
Hypertension	1.517 (1.372–1.678)	**<0.001**
Arthritis	1.180 (1.056–1.320)	**0.004**
Asthma	0.695 (0.564–0.857)	**<0.001**
Diabetes	1.515 (1.323–1.735)	**<0.001**
HIV positive/AIDS	2.017 (1.239–3.282)	**0.005**

* Adjusted analysis for age, gender and smoking in determining the association between bone loss and systemic inflammatory conditions with *p* ≤ 0.05. Bold denotes statistical significance.

## Data Availability

The data presented in this study are available on request from the corresponding author.
